# Harnessing Artificial Intelligence for Precision Cardiovascular Medicine

**DOI:** 10.7759/cureus.97837

**Published:** 2025-11-26

**Authors:** Arijita Banerjee, Pradosh Kumar Sarangi

**Affiliations:** 1 Physiology, Indian Institute of Technology, Kharagpur, West Bengal, IND; 2 Radiodiagnosis, All India Institute of Medical Sciences, Deoghar, Rampur, IND

**Keywords:** arrythmia, cardiac imaging, deep learning, electrocardiography (ecg), electronic health records, machine learning, neural networks

## Abstract

Artificial intelligence (AI) has revolutionized cardiology diagnostic capabilities by improving precision, effectiveness, and prompt identification of various cardiac diseases. AI subfields include machine learning, deep learning, and cognitive computing. Machine learning can be supervised, unsupervised, or reinforcement learning. Support vector machines (SVM), deep learning, and artificial neural networks (ANN) are commonly used in the medical field for handling large and complex data. ANNs perform better than SVMs in evaluating electrocardiogram (ECG) data, while SVMs are used for disease stratification. AI-driven diagnostic tools have transformed the interpretation of ECGs, echocardiograms, cardiac imaging, and other diagnostic modalities, leading to better patient outcomes and more accurate clinical decision-making. AI has shown promise in identifying and describing coronary artery disease, with machine learning models training on cardiac CT images. Non-invasive AI tools, like HeartFlow, help patients with cardiac autonomic dysfunction (CAD) with treatment planning and decision-making. AI systems often require access to sensitive patient data, raising concerns about privacy, data security, and consent. Additionally, using patient data without clear ethical oversight may erode public trust. Transparency in how AI tools use and protect data is essential to maintain ethical standards in cardiovascular medicine.

## Introduction and background

A new era of cardiology diagnostic capabilities has been brought about by artificial intelligence (AI), which has greatly improved the precision, effectiveness, and prompt identification of a variety of cardiac diseases. Machine learning (ML), deep learning (DL), and cognitive computing are some of the subfields of artificial intelligence. Based on whether external supervision is present or not during training, machine learning can be divided into three categories: supervised learning, unsupervised learning, and reinforcement learning [1-3].

Using a set of samples from a known class, supervised learning - also referred to as supervised training - is the process of adjusting a classifier's parameters to attain the desired performance. AI plays a crucial role in medicine by processing data by simulating the human brain. It can recognise, process, integrate, and analyse vast amounts of medical data. These days, support vector machines (SVM), DL, and artificial neural networks (ANN) are the most often used algorithms in the medical field. When dealing with large and complex data, including nonlinear relations, ANNs and SVMs could be extremely helpful. When evaluating electrocardiogram (ECG) data, ANNs perform better than SVMs, whereas SVMs are used for disease stratification. For instance, echocardiograms (ECGs) processed by AI algorithms are currently used by clinicians to diagnose various cardiopulmonary conditions like atrial fibrillation, hypertrophic cardiomyopathy, anaemia, and pulmonary hypertension [4-7].

**Figure 1:**
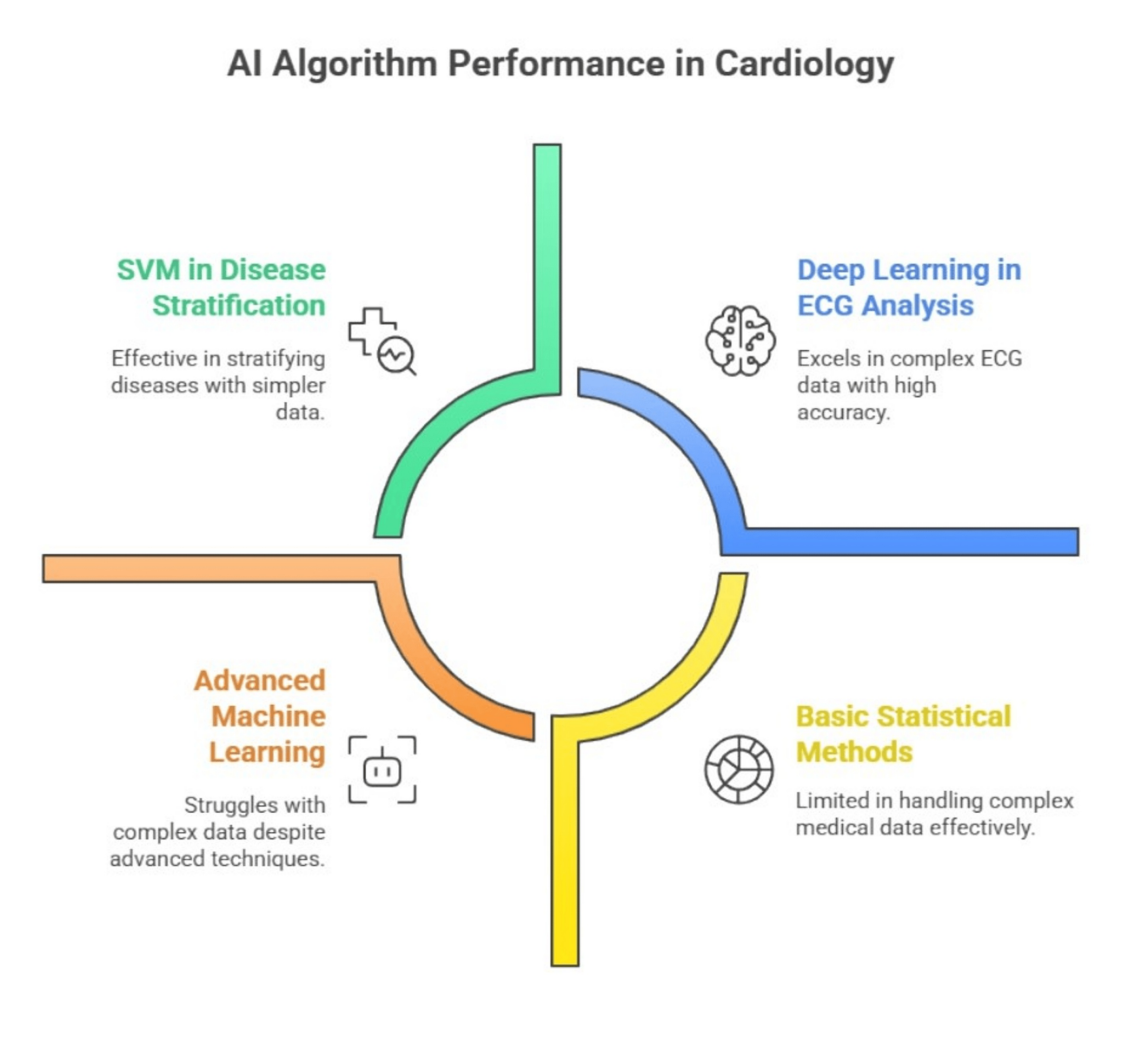
Artificial intelligence algorithms in cardiology Image created by the authors for illustrative purposes.

By providing pre-diagnosis, fixing clinician errors, and preventing misdiagnosis, approved and verified algorithms may lessen the cognitive load on clinicians once they are implemented in the clinic. The interpretation of ECGs, echocardiograms, cardiac imaging, and other diagnostic modalities has been transformed by AI-driven diagnostic tools, leading to better patient outcomes and more accurate clinical decision-making for a range of cardiac conditions. ECG analysis has advanced significantly thanks to AI-powered algorithms, allowing for quick and precise interpretation of these vital tests, as shown in Figure 1. AI systems can precisely identify certain conditions, such as conduction abnormalities and arrhythmias (like atrial fibrillation), which helps with prompt diagnosis and suitable treatment plans. Businesses such as AliveCor (Mountain View, CA, US), for example, have created AI-based algorithms that can identify atrial fibrillation from smartphone ECG recordings, enabling early intervention and lowering the risk of stroke [8,9].

AI has had a major impact on echocardiography, allowing for a more accurate and thorough examination of the anatomy and function of the heart. The accuracy of identifying and characterising different cardiac pathologies has increased thanks to AI-driven advancements in cardiac imaging such as MRI and CT scans. Early diagnosis of conditions like pulmonary artery hypertension (PAH) is greatly aided by AI-based technologies. To identify and forecast the course of PAH, researchers have created AI algorithms that analyse cardiac MRI images. These instruments aid in the detection of minute alterations in cardiac and pulmonary circulation, enabling prompt treatment and better patient care [10,11].

Significant promise has also been shown by AI in the identification and description of coronary artery disease. Accurate detection and measurement of coronary artery stenosis is achieved by training machine learning models on large datasets of cardiac CT angiography images. To help patients with CAD with treatment planning and decision-making, non-invasive AI-based tools have been developed by companies such as HeartFlow (Mountain View, CA, US). These tools generate customised 3D models of coronary arteries to evaluate the functional significance of blockages. AI-based predictive models forecast heart failure exacerbations by utilising a variety of data sources such as imaging and patient records [12,13].

By facilitating proactive interventions and individualised management plans, these models help clinicians identify high-risk individuals, optimise treatment strategies, and improve patient outcomes. In order to lessen the burden of heart failure readmissions and enhance patient outcomes, clinicians can proactively identify patients who are at increased risk of hospitalisation, execute specific approaches, and develop optimal approaches to therapy by utilising AI to analyse a variety of patient data, such as clinical parameters, biomarkers, and imaging results. The main areas of cardiovascular practice and research where artificial intelligence is anticipated to become increasingly prevalent over the next 10 years are thoroughly reviewed in this paper.

## Review

Methodology

A comprehensive literature search was performed across major electronic databases, including PubMed, Scopus, Web of Science, and IEEE Xplore, to identify studies evaluating the use of artificial intelligence (AI) in the diagnosis of cardiovascular diseases (CVDs). The search was carried out for articles published between January 2013 and September 2025, reflecting the period of rapid advancement in AI applications within cardiovascular medicine, as shown in Table 1. Keywords and Medical Subject Headings (MeSH) terms used in various combinations included “artificial intelligence,” “machine learning,” “deep learning,” “electrocardiogram,” “echocardiography,” “cardiac imaging,” “cardiac arrhythmia,” “valvular heart disease,” “autonomic dysfunction,” and “cardiovascular disease diagnosis.”

**Table 1:** PRISMA summary of the study selection process PRISMA: Preferred Reporting Items for Systematic reviews and Meta-Analyses

Stage	Description	Number of Records
Identification	Records identified through database searches (PubMed, Scopus, Web of Science, IEEE Xplore)	2,346
	Additional records identified through manual reference searching	42
Screening	Records after duplicates removed	1,734
	Records excluded after title/abstract screening	1,212
Eligibility	Full-text articles assessed for eligibility	522
	Full-text articles excluded (reviews, no human data, insufficient metrics, etc.)	401
Included	Studies included in qualitative synthesis	121
	Studies included in quantitative analysis (with diagnostic accuracy data)	84

Inclusion criteria were: (1) original peer-reviewed studies evaluating AI, ML, or DL models for diagnosing or predicting cardiovascular diseases; (2) studies utilizing clinical data such as ECG, echocardiography, CMR, CT, or wearable device signals; (3) articles published in English; and (4) studies reporting quantitative performance metrics such as accuracy, sensitivity, specificity, or area under the curve (AUC). Exclusion criteria were: (1) reviews, editorials, conference abstracts without full text, or case reports; (2) studies focusing solely on AI model development without validation on human clinical datasets; (3) papers unrelated to CVD diagnosis (e.g., AI in treatment optimisation or administrative use); and (4) non-English publications.

AI-aided CVD diagnosis

For CVDs, timely treatment, identification, and detection are crucial for reducing the rate at which the disease progresses to more advanced stages and enhancing overall results. The gold standards for diagnosing certain CVDs, including dilated cardiomyopathy, aortic stenosis, and ventricular dysfunction, are frequently cardiac magnetic resonance imaging (ECG) and cardiac magnetic resonance imaging (CMR). Early diagnosis of CVDs is made more difficult by the high cost and technical expertise needed to use these extra tools, which may not be appropriate as screening tools for the general public. Even in places with little funding, ECG is a cheap, accessible, and easy-to-use auxiliary test. The ECG has long been a useful diagnostic tool for CVDs. However, the degree of experience and expertise of the clinician determines how they interpret the ECG. Additionally, the raw ECG waveform has a huge number of data points that are hard for clinicians to evaluate, which makes it difficult to fully utilise its advantage. In order to identify CVDs, we will examine the most recent developments in the application of AI technology to standard 12-lead ECG [14,15].

Cardiac Arrhythmias

The generation of a normal cardiac rhythm and the transfer of electrical impulses throughout the myocardium depend on the integration of ion channels and transporters as well as the intrinsic automaticity of myocardial cells. Patients suffer from cardiac arrhythmias when either the conduction of action potentials or the normal electrophysiological process of impulse generation is disturbed. Atrial fibrillation (AF), the most common type, results in ineffective contractions due to its rapid and irregular electrical signals in the atria. Shortness of breath, fatigue, palpitations, and an increased risk of stroke are symptoms that AF patients present with. During ECG recording, patients with AF frequently exhibit a normal sinus rhythm, which could result in an underdiagnosis. But once AF develops, the heart's structure begins to alter [15]. In order to predict AF, a deeply trained neural network may be able to detect subtle changes in the normal sinus-rhythm ECGs. Using a standard 10-second, 12-lead ECG, Attia et al. have implemented a CNN to identify patients with AF during normal sinus rhythm. The accuracy and correlation of AI-ECG and CHARGE-AF (Cohorts for Heart and Ageing Research in Genomic Epidemiology-Atrial Fibrillation) scores in forecasting future AF risk were then compared by Hokken et al. [5]. Studies like the Apple Heart Study, Fitbit Heart Study, and Huawei Heart Study have shown that smartwatches can identify irregular pulses and use ECG patch monitoring to confirm the diagnosis of AF. With nearly 650,000 ECGs, the Mayo Clinic developed the first AI-ECG algorithm to predict paroxysmal AF on ECGs from patients in sinus rhythm. Additionally, patients who experienced an embolic stroke of unknown cause - in which case silent underlying AF is frequently suspected as the cause - were studied for the use of AI-ECG for AF estimation. With 96% accuracy, smartphone-based algorithms have been able to differentiate PVCs from sinus rhythm, PACs, and AF. Alive Cor Kardia Monitor and the Apple Watch are two mHealth devices that have been tested for sudden cardiac death in comparison to traditional 12-lead ECGs in children [16-20].

Valvular Heart Defects

Valvular heart disease (VHD) remains challenging due to variability in presentation, progression, and the resources needed for accurate diagnosis and monitoring. AI and machine learning (ML) are increasingly applied in four main domains: screening, assessment/severity classification, risk prediction, and treatment planning/phenotyping. AI applied to ECGs and digital or AI‑enabled stethoscopes can detect valvular defects earlier or in settings where echocardiography is not immediately available. One study used an AI stethoscope on 514 patients (304 training, 210 testing) to identify left‑sided VHD; the area under the receiver operating characteristic (ROC) for composite VHD was ~0.85 in training, ~0.76 in testing; sensitivity ~70%, specificity ~74%. Another study found that AI via a digital stethoscope detected 94.1% of cases of VHD, versus 41.2% by a traditional stethoscope, though with somewhat lower specificity. Algorithms trained on ECG also showed ~69‑79% correct prediction of future regurgitant VHD in some reports [21-24].

**Figure 2:**
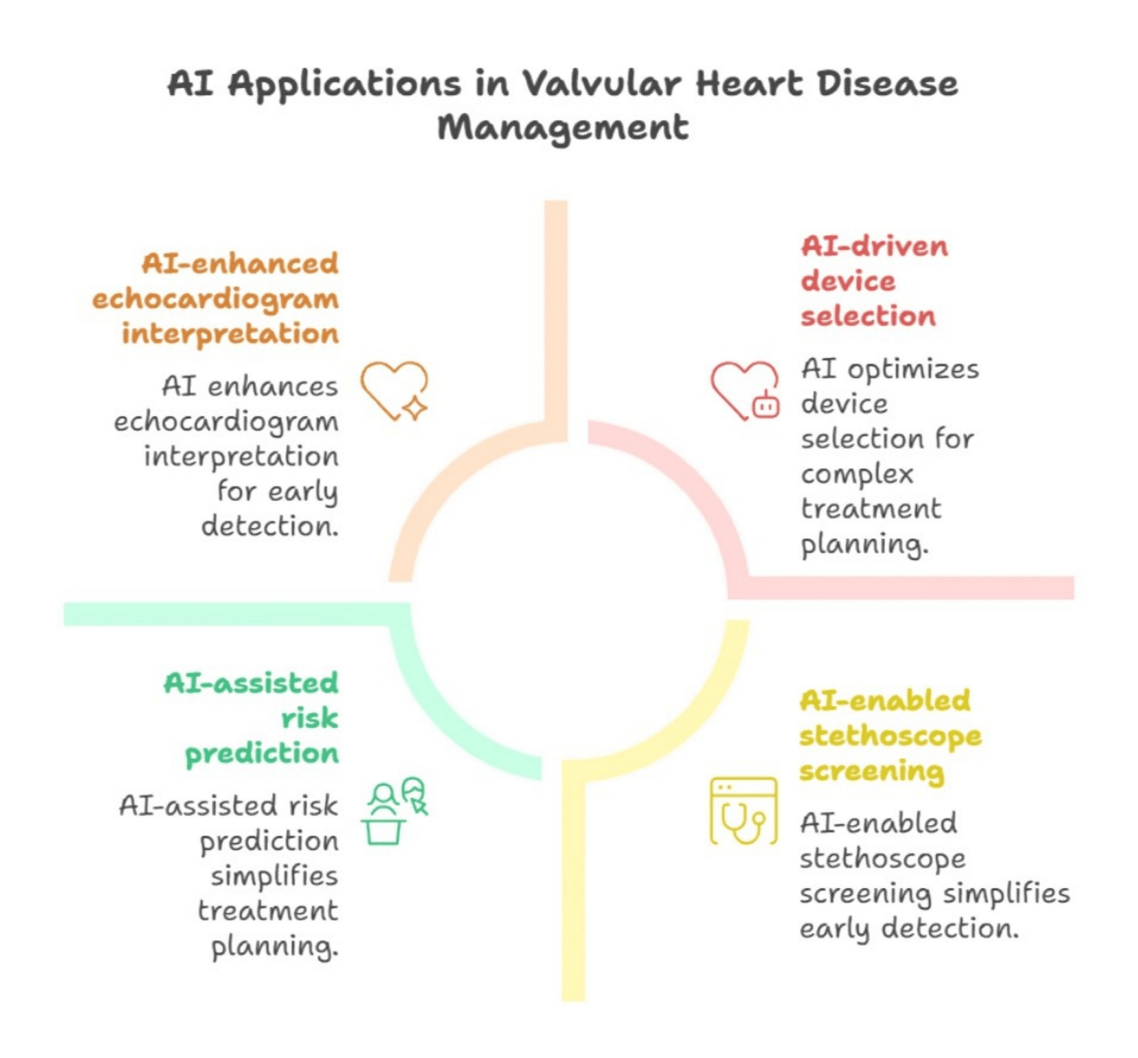
Artificial intelligence in valvular heart disease Image created by the authors for illustrative purposes.

AI models are being used to help with interpreting echocardiograms: view recognition, segmentation of valve structures, automating calculation of gradients, flows, volumes, and global longitudinal strain, etc. In a case series on bicuspid aortic valves, for example, AI tools were used to automatically estimate pressure gradients and velocity time integrals, reconstruct 3D valve anatomy, compute left ventricular volumes, ejection fraction (EF), and strain to guide whether repair vs replacement was indicated. AI helps stratify which patients with VHD are likely to progress more rapidly or suffer complications, enabling better timing for interventions, as shown in Figure 2. For instance, in a DL model combining multimodal echo data from 8,472 patients with aortic valve disease, researchers could classify stenosis severity and predict progression over 24 months with ~91.7% accuracy and AUC ≈ 0.918. AI is also being explored for helping device selection (e.g. transcatheter valves), procedural planning, predicting risk of complications, and optimising follow‑up after interventions [25-27].

Cardiac Autonomic Dysfunction

Cardiac autonomic dysfunction (CAD), including cardiac autonomic neuropathy (CAN), reflects impaired autonomic regulation of heart rate, often seen in diabetes, hypertension, neurodegenerative disorders, depression, post‑viral syndromes, etc. AI/ML methods are increasingly used to improve detection, classification, prognosis, and monitoring of such dysfunction [28-31].

One notable recent study is the Silesia Diabetes‑Heart Study, which used AI on retinal fundus images to classify CAN in patients with diabetes. Using ~2,275 retinal images from 229 patients, a model based on ResNet‑18 correctly identified 93% of patients with CAN (sensitivity) and 89% without CAN (specificity) for binary classification; AUCROC was 0.87. For the more severe (definite or severe CAN, “dsCAN”) vs non‑dsCAN grouping, sensitivity was lower (78%) but specificity was very high (93%), AUCROC 0.94. Another study focussed on ECG‑based detection: “Artificial intelligence‑enhanced electrocardiogram analysis for identifying cardiac autonomic neuropathy in patients with diabetes” used 12‑lead, 10‑second ECGs and ML/motif & discord extraction + long short‑term memory (LSTM) models. Among 205 diabetic patients (100 with any CAN; the subset had definite or severe CAN, eCAN or dsCAN), the best model achieved an accuracy of ~0.92, F1 score 0.92, recall (sensitivity) ≈ 0.94, precision ~0.91, and AUC ~0.93 for dsCAN detection [32-34].

In Type 2 diabetes mellitus, heart rate variability (HRV) during rest and after orthostatic challenge was analysed using ML algorithms (the Classification and Regression Tree model, among others). The CART model achieved 84.04% accuracy, sensitivity ~89.51%, specificity ~66.67%, with AUC ~0.78 in distinguishing diabetics with autonomic dysfunction vs controls. Resting HRV alone had lower performance (accuracy ~75.1%, specificity only ~39.2%, AUC 0.63). In post‑COVID‑19 syndrome, cardiovascular dysautonomia was observed in ~15.2% of recovered patients (orthostatic hypotension ~13%, postural orthostatic tachycardia ~2.2%). The HRV (RMSSD) was significantly lower in post‑COVID patients vs healthy controls (13.9 ±11.8 ms vs 19.9 ±19.5 ms, p = 0.01), and ML (multi‑layer perceptron) was best at distinguishing between groups [35-38].

Use of AI in Cardiac Imaging for Diagnosing Cardiovascular Diseases

Advances in medical imaging - echocardiography, cardiac computed tomography (CT), cardiac magnetic resonance (CMR), nuclear imaging - have yielded enormous datasets. AI methods, especially machine learning (ML) and deep learning (DL), are increasingly applied to extract clinically useful information, improve diagnostic accuracy, speed, and reproducibility. AI systems are used to segment cardiac structures (chambers, myocardium, valves), delineate borders, quantify metrics like ventricular volumes, EF, wall thickness, strain, and scar extent. This reduces inter‑observer variability and saves time. For example, a platform called IntelliCardiac, trained on the public ACDC dataset, achieved ~92.6% segmentation accuracy and ~98% classification accuracy among five diagnostic categories (dilated cardiomyopathy, hypertrophic cardiomyopathy, myocardial infarction, right ventricular abnormality, and no disease) [39-41].

AI applied to coronary CT angiography (CTA), coronary artery calcium (CAC) scoring, and nuclear imaging can quantify plaque burden, plaque composition, and functional metrics. ML models combining imaging biomarkers with clinical data have been shown to improve risk prediction of coronary artery disease and adverse events. AI can detect structural heart disease (valves and wall motion abnormalities) from ECGs, or “hidden” imaging signs, potentially enabling screening. A recent example is a tool called EchoNext, which uses ECG + AI to identify patients at high risk of structural heart disease who should undergo echocardiography. In a comparative test, EchoNext achieved ~77% accuracy vs ~64% for cardiologists when reading ECGs in detecting such a disease.

AI helps accelerate imaging processes (e.g. faster MRI scan protocols), denoise images, reduce radiation exposure in CT, and assist less‐experienced operators in image acquisition. AI was found to enhance image quality, speed imaging, and reduce radiation exposure, with sensitivity and specificity comparable to or exceeding clinicians in many studies [42-44].

Discussion

Artificial intelligence (AI) is revolutionising cardiology by enhancing diagnostic accuracy, treatment personalisation, and patient outcomes. Through machine learning and deep learning algorithms, AI can analyse vast datasets, such as electrocardiograms (ECGs), echocardiograms, cardiac imaging, and electronic health records, to detect subtle patterns that may escape human observation. This enables early diagnosis of cardiovascular diseases like arrhythmias, heart failure, and coronary artery disease. AI-driven tools assist clinicians in risk prediction, patient monitoring, and decision-making, leading to more precise and timely interventions. For example, AI algorithms can predict the likelihood of cardiac events by integrating clinical, genetic, and lifestyle data. In imaging, AI improves efficiency by automating image segmentation and interpretation, reducing workload and human error. Moreover, wearable AI technologies continuously monitor heart health, supporting preventive cardiology and remote care. Overall, AI bridges the gap between data and clinical practice, promoting evidence-based, individualised care. Its integration into cardiology not only enhances diagnostic and prognostic accuracy but also optimises healthcare resources, paving the way for more proactive and predictive cardiovascular medicine [45].

Artificial Intelligence (AI) is revolutionising cardiovascular medicine, offering advances in diagnosis, risk prediction, imaging interpretation, and clinical decision-making. Despite its promise, the integration of AI into routine cardiovascular care faces significant challenges and limitations that must be addressed before widespread clinical adoption.

Challenges and Limitations of Artificial Intelligence in Cardiovascular Medicine

Data quality and availability: AI systems require large, high-quality datasets for training. In cardiovascular medicine, such datasets may be limited, especially in rare diseases or in underserved populations. Many models are trained on retrospective, single-centre datasets, which lack diversity in terms of demographics, disease spectrum, and imaging protocols. As a result, the trained models may not generalise well across institutions or patient populations [46].

Bias and inequity:* *AI systems are susceptible to biases inherent in training data. For example, if a model is trained predominantly on data from one ethnic group, it may perform poorly in others. This raises serious concerns about fairness and equity. In cardiovascular care, where disease prevalence and presentation differ by age, sex, and ethnicity, biased AI models risk perpetuating healthcare disparities rather than alleviating them [47].

Lack of interpretability:* *Many AI models, particularly deep learning networks, function as “black boxes.” They produce results without providing transparent reasoning. This lack of explainability is a major limitation in clinical decision-making, where understanding the rationale behind a diagnosis or recommendation is crucial for clinician trust and patient safety. Clinicians are unlikely to rely on AI without confidence in how conclusions were reached [48].

Regulatory and legal challenges:* *AI tools in healthcare face unclear regulatory pathways. Determining who is accountable when AI systems fail - developers, clinicians, or institutions - is legally complex. Furthermore, regulatory bodies like the FDA are still evolving frameworks to evaluate and approve AI-driven tools, especially those that adapt and learn over time. Without clear guidelines, integration into clinical workflows is limited [49].

Integration into clinical workflows:* *AI solutions often exist in research or pilot settings but are not integrated into electronic health records (EHRs) or hospital systems. For clinicians already burdened with time constraints, introducing AI tools that require separate platforms or training may be impractical. Seamless integration into existing clinical workflows remains a significant barrier [50].

Validation and reproducibility: Many AI studies in cardiology report high performance in development cohorts but fail to replicate results in external validation cohorts. Without rigorous, prospective validation, AI models cannot be safely deployed in clinical practice. Lack of standardisation in performance metrics also complicates comparisons across studies [51].

Ethical and privacy concerns: AI systems often require access to sensitive patient data, raising concerns about privacy, data security, and consent. Additionally, using patient data without clear ethical oversight may erode public trust. Transparency in how AI tools use and protect data is essential to maintain ethical standards in cardiovascular medicine [52].

A cardiology service evaluating an artificial intelligence (AI) tool should adopt a structured framework that ensures clinical safety, ethical integrity, and regulatory compliance. Governance begins with establishing clear accountability through an AI oversight committee that includes clinicians, data scientists, and legal experts to ensure the tool aligns with clinical standards and approved regulatory frameworks. Validation should involve rigorous assessment of the tool’s performance, including technical accuracy, clinical relevance, and external generalizability using local patient data to confirm reliability. Continuous monitoring is essential to detect performance drift, audit usage, and maintain a feedback loop for clinicians to report anomalies or errors. Informed consent and transparency are equally vital; patients should be made aware when AI contributes to their diagnosis or management and understand its role and limitations. Finally, strong data protection measures must be in place, including compliance with data privacy regulations, such as the General Data Protection Regulation (GDPR), encryption of patient information, and anonymisation of datasets used for model training and evaluation. Together, these practices ensure that AI integration in cardiology is safe, effective, and ethically sound [48-51].

## Conclusions

Artificial intelligence has emerged as a transformative force in cardiovascular medicine, offering unprecedented capabilities in diagnosis, risk assessment, and personalised treatment. As technology advances, the collaboration between AI systems and clinicians will be crucial to ensure ethical use, transparency, and patient safety, ultimately shaping a smarter, more precise, and preventive future for cardiovascular healthcare. While artificial intelligence holds transformative potential in cardiovascular medicine, several limitations hinder its safe and equitable implementation. Addressing issues of data quality, bias, explainability, legal liability, workflow integration, and ethical concerns is essential. Future efforts should focus on robust validation, transparent model development, and inclusive datasets to ensure that AI in cardiology enhances, not replaces, clinical judgment, improves outcomes, and serves all patient populations equitably.
